# Abdominal compartment syndrome due to extremely elongated sigmoid colon and rectum plus fecal impaction caused by disuse syndrome and diabetic neuropathy: a case report and review of the literature

**DOI:** 10.1186/s13256-020-02566-8

**Published:** 2020-11-13

**Authors:** Daisuke Usuda, Kohei Takanaga, Ryusho Sangen, Toshihiro Higashikawa, Shinichi Kinami, Hitoshi Saito, Yuji Kasamaki

**Affiliations:** 1 Department of General Medicine, Kanazawa Medical University Himi Municipal Hospital, 1130 Kurakawa, Himi-shi, Toyama-ken 935-8531 Japan; 2 Department of Infectious Diseases, Kanazawa Medical University, Uchinada-machi, Ishikawa-ken 920-0293 Japan; 3 Department of Geriatric Medicine, Kanazawa Medical University Himi Municipal Hospital, Himi-shi, Toyama-ken 935-8531 Japan; 4 Department of General and Digestive Surgery, Kanazawa Medical University Himi Municipal Hospital, Himi-shi, Toyama-ken 935-8531 Japan

**Keywords:** Abdominal compartment syndrome, Intra-abdominal hypertension, Elongated sigmoid colon, Fecal impaction, Diabetic neuropathy, Operation

## Abstract

**Background:**

Abdominal compartment syndrome (ACS) is defined as a sustained raised level of intra-abdominal pressure more than 20 mmHg with or without abdominal perfusion pressure less than 60 mmHg and the development of new end-organ failure. Abdominal surgery, major trauma, volvulus, ileus, distended abdomen, fecal impaction, acute pancreatitis, liver dysfunction, sepsis, shock, obesity, and age have all been reported as risk factors. Herein, we report the severest known case of ACS due to extremely elongated sigmoid colon and rectum plus fecal impaction caused by disuse syndrome and diabetic neuropathy, together with a brief review of the literature.

**Case presentation:**

A 48-year-old Asian man suffering from shock was transported by ambulance to our hospital. His medical history included hypoglycemic encephalopathy sequelae, disuse syndrome, type 2 diabetic neuropathy, and constipation. He recovered consciousness in the ambulance, and his physical examination as well as laboratory findings were normal. X-ray and dynamic computed tomography revealed a thickened gut wall, and an extremely dilated sigmoid colon and rectum filled with a massive amount of stool as well as gas, compressing other intra-abdominal organs. We diagnosed the patient with transient vasovagal syncope, together with ACS, due to extremely elongated sigmoid colon and rectum plus fecal impaction, caused by anorectal disturbance derived from disuse syndrome and diabetic neuropathy. We first repeated stool extraction for bowel decompression and he subsequently became symptom-free, after which we performed a colostomy on the 28th hospital day. The postoperative course was uncomplicated, and he was discharged on the 44th hospital day.

**Conclusions:**

Clinicians need to keep ACS in mind as a differential diagnosis and perform careful and detailed examination when encountering patients presenting with symptoms or risk factors of ACS. In addition, they need to precisely diagnose ACS and perform optimal treatment without delay.

## Background

Intra-abdominal hypertension (IAH)/abdominal compartment syndrome (ACS) are rare but potentially morbid diagnoses [1]. ACS was first reported in 1984, and its pathophysiology was described as resulting from IAH [2]. ACS is defined as a sustained raised level of intra-abdominal pressure (IAP) more than 20 mmHg with or without abdominal perfusion pressure less than 60 mmHg and the development of new end-organ failure; its reported risk factors are shown in Table 1 [3–9]. Among these, there are several reports of ACS associated with or caused by fecal impaction [7–9]. In addition, IAH is defined as a sustained elevated level of IAP greater than 12 mmHg. As far as we know, we report herein the severest known case of ACS due to extremely elongated colon and rectum plus fecal impaction caused by disuse syndrome and type 2 diabetic neuropathy, together with a brief review of literature.

**Table 1.** Cause of abdominal compartment syndrome and intra-abdominal hypertension

Condition	ACS	Primary cause of IAH	Secondary cause of IAH
Cause	Abdominal surgery	Trauma	Extra-abdominal causes
	Major trauma	Diseases of the abdominopelvic region	Sepsis
	Volvulus	Pancreatitis	Burns
	Ileus	Abdominal surgery	
	Distended abdomen		
	Fecal impaction		
	Acute pancreatitis		
	Liver dysfunction		
	Sepsis, shock		
	Obesity		
	Age		

*ACS* abdominal compartment syndrome, *IAH* intra-abdominal hypertension

## Case presentation

A 48-year-old Japanese man suffering from shock was transported by ambulance to our hospital. He recovered consciousness in the ambulance. His medical history included hypoglycemic encephalopathy sequelae, which led to poor performance status, namely disuse syndrome, type 2 diabetic neuropathy, constipation, and transient vasovagal syncope after defecation. He was treated with sitagliptin phosphate, voglibose, and a laxative, and did not take any medicines that could slow bowel movements, such as antidepressive drugs. The patient was unemployed and did not have any food or drug allergies. His family history contained nothing of note; specifically, there was no family history of any immunodeficiency disorder or other congenital anomalies.

**Fig. 1:**
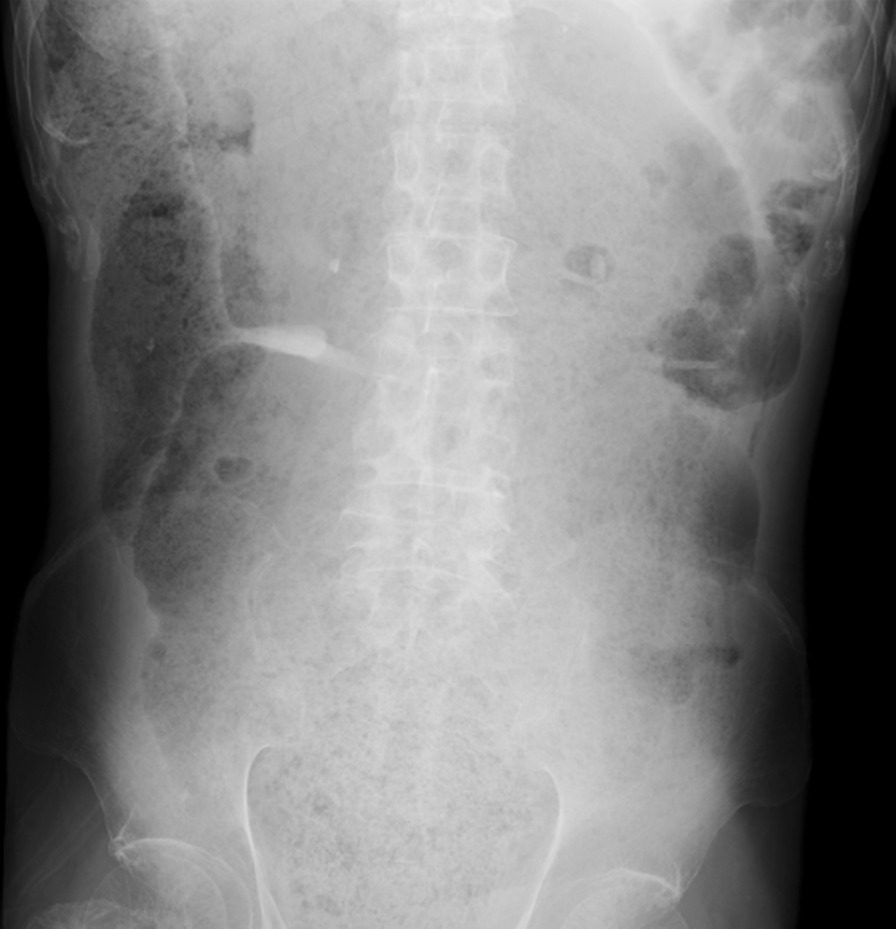
An abdominal X-ray on admission revealed a thickened gut wall and extremely dilated sigmoid colon and rectum filled with a massive amount of stool as well as gas

**Fig. 2:**
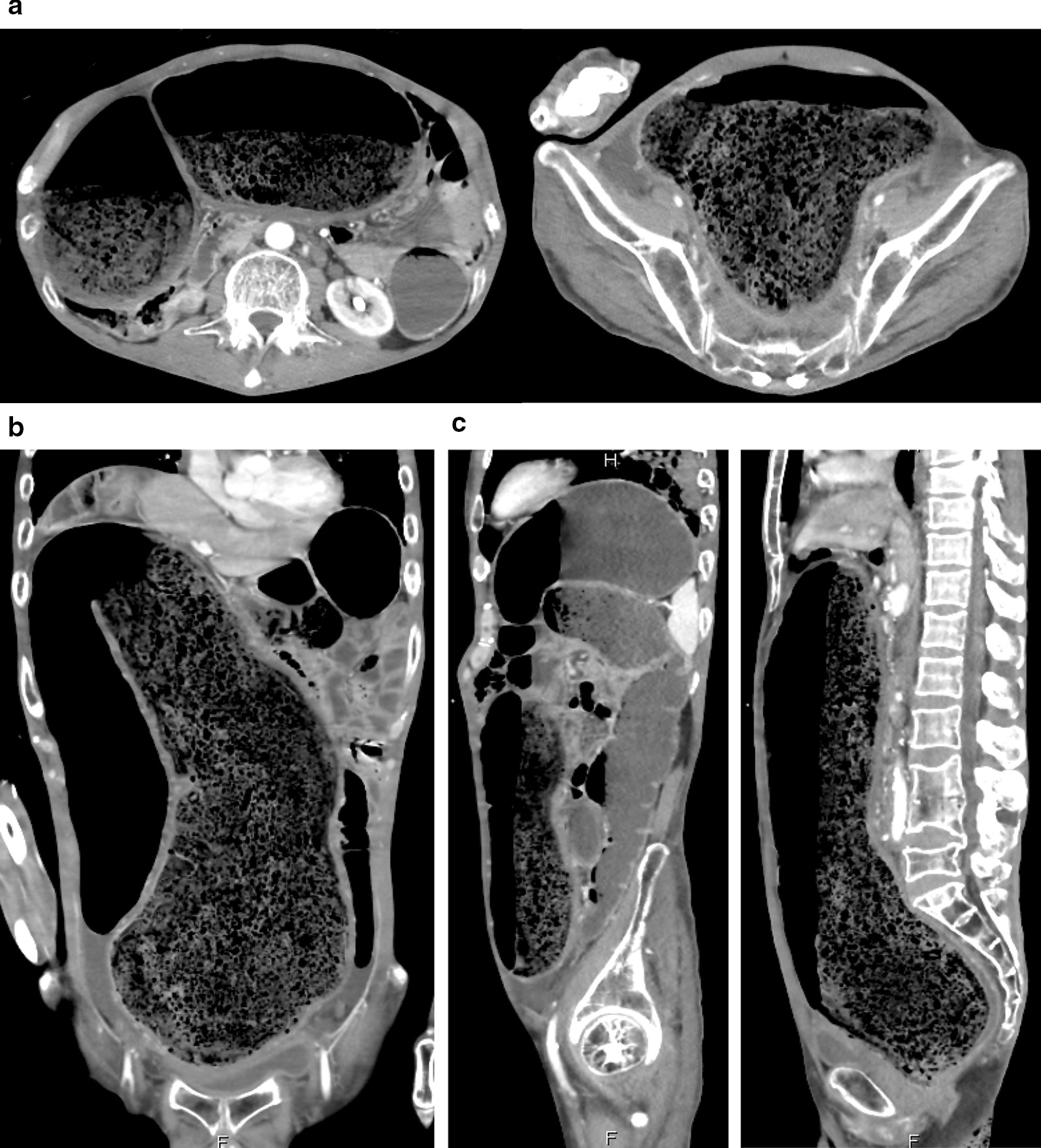
Arterial phase of abdominal dynamic computed tomography (**a** transverse plane, **b** coronal plane, **c** sagittal plane) on admission. Abdominal dynamic computed tomography of the abdomen revealed a thickened gut wall and extremely dilated sigmoid colon and rectum filled with a massive amount of stool as well as gas, compressing other intra-abdominal organs

The patient was 175 cm tall and weighed 47 kg. His vital signs were abnormal, with blood pressure of 82/52 mmHg, heart rate of 67 beats per minute, body temperature of 35.1 °C, oxygen saturation of 97% in ambient air, and respiratory rate of 16 per minute; his Glasgow Coma Scale score was 15 (E4V5M6). Nothing abnormal was detected upon physical examination. The patient’s laboratory findings were normal, including complete blood count, biochemistry, casual blood glucose, ammonia, and urine tests. An abdominal X-ray and arterial-phase dynamic computed tomography revealed a thickened gut wall and an extremely dilated sigmoid colon and rectum filled with a massive amount of stool as well as gas, compressing other intra-abdominal organs (Figs. 1 and 2, respectively). On the other hand, no apparent volvulus or peritonitis was observed. We diagnosed the patient with transient vasovagal syncope, together with abdominal compartment syndrome (ACS), due to extremely elongated sigmoid colon and rectum plus fecal impaction, caused by anorectal disturbance derived from disuse syndrome and diabetic neuropathy.

**Fig. 3:**
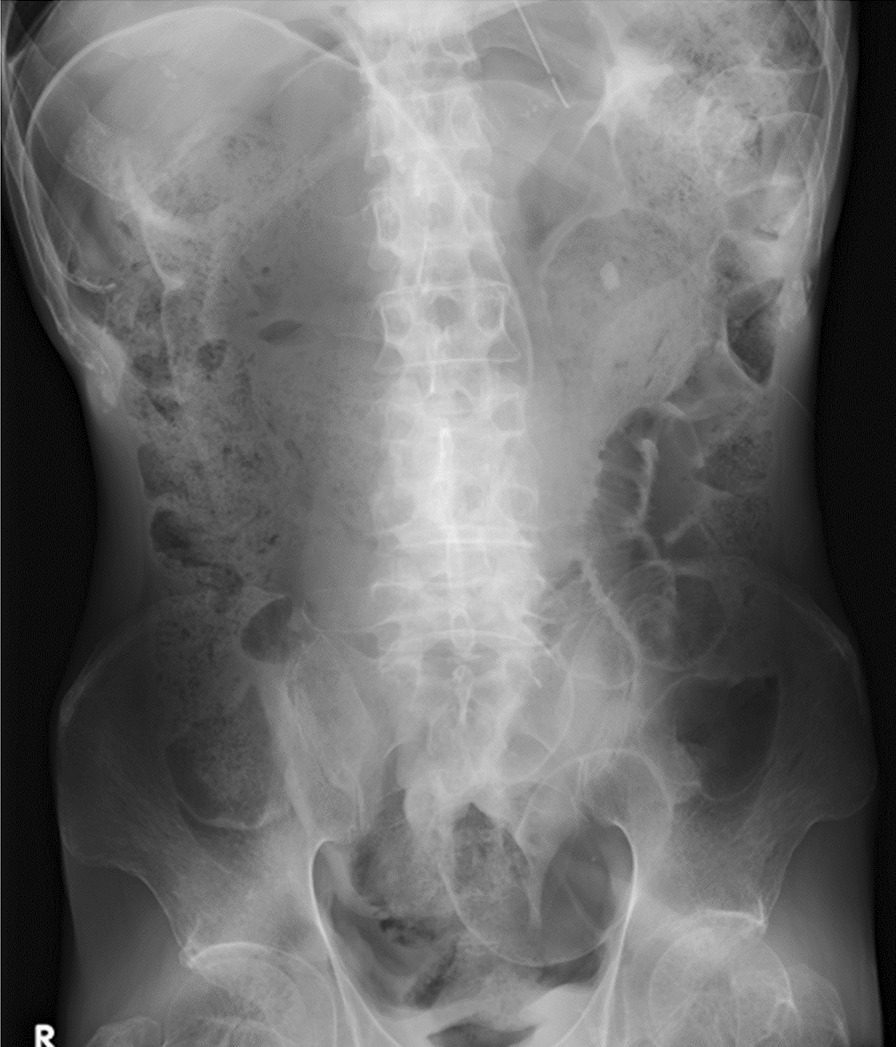
An abdominal X-ray on the 41st hospital day revealed relief of dilated sigmoid colon and rectum together with massive amount of stool

After discussion with his family, we first repeated stool extraction for bowel decompression, and he subsequently became symptom-free, after which we performed a colostomy on the 28th hospital day to prevent a recurrence. The operation was performed after the patient was symptom-free in order to reduce the risk of intraoperative or postoperative complications. The postoperative course was uncomplicated, and an abdominal X-ray on the 41st hospital day revealed relief of dilated sigmoid colon and rectum together with massive amount of stool (Fig. 3). He transferred hospitals on the 44th hospital day in order to continue treatment, and we did not follow him afterwards.

## Discussion and conclusions

We found no other cases of ACS showing a more severe elongated colon among patients who had disuse syndrome and type 2 diabetic neuropathy. Therefore, this is the severest known case of ACS due to extremely elongated sigmoid colon and rectum plus fecal impaction. Recent developments related to IAH/ACS and clinical practice guidelines were published in 2013 [10]. In general, IAH may be induced by any of several intra-abdominal or extra-abdominal conditions, reduced abdominal wall compliance, or intra-abdominal pathologies (of either the peritoneal space or parenchymatous organs); its mechanism is mucosal and submucosal tissue hypoperfusion, which causes considerable damage to the intestinal cells, potentially resulting in bacterial translocation, endotoxin release, or sepsis [11, 12]. Among these, most commonly, intra-abdominal infections and/or sepsis and severe trauma or burns are predisposed to lead to IAH [12]. IAH is caused by decreased perfusion of the kidneys, abdominal viscera, splanchnic organs, central nervous system, and possible difficulties with ventilation and maintenance of cardiac output, and can lead to multiple-organ dysfunction [11, 13, 14]. IAH can be classified as either primary or secondary, and those causes are shown in Table 1 [15]. ACS is the end point of a process whereby massive interstitial swelling in the abdomen or rapid development of a space-filling lesion in the abdomen (such as ascites or a hematoma) leads to pathologically increased pressure [13]. Massive fecal impaction leading to surgical catastrophes has rarely been reported, and neglected fecal impaction may lead to megarectum causing ACS and colorectal obstruction, perforation, or necrosis [16]. In this case, diabetic neuropathy and disuse syndrome led to fecal impaction and distended abdomen; they then caused secondary IAH and ACS.

Measurement of intravesical pressure is the gold standard for diagnosing IAH, as well as measurement of bladder pressure: pressure greater than 12 mmHg is consistent with IAH, and greater than 25 mmHg is consistent with ACS [1, 17]. There are an increasing number of techniques that allow us to measure intra-abdominal pressure (IAP) at the bedside [18]. IAP should be measured at end-expiration, with the patient in the supine position, after ensuring that there is no abdominal muscle activity [18]. The level where the mid-axillary line crosses the iliac crest is the recommended zero reference for the transvesical IAP measurement; marking this level on the patient also increases reproducibility [18]. In children, normal IAP in mechanically ventilated patients is approximately 7 ± 3 mmHg [19]. As an IAP of 10–15 mmHg has been associated with organ damage in children, an IAP greater than 10 mmHg should be considered IAH in these patients [19]. Moreover, as ACS may occur in children at an IAP lower than 20 mmHg, any elevation in IAP greater than 10 mmHg associated with new organ dysfunction should be considered ACS in children until proven otherwise [19]. In this case, IAP was not measured, but IAH and ACS were strongly suspected based on the patient’s symptoms as well as physical examinations and imaging investigations.

Medical management of critically ill patients with raised IAP should be instigated early to prevent further organ dysfunction and to avoid progression to ACS [20]. Appropriate actions should be taken when IAP increases above 15 mmHg, especially if pressures reach 20 mmHg or greater with new-onset organ failure [21]. Therapy for IAH/ACS consists of five treatment "columns": intraluminal evacuation, intra-abdominal evacuation, improvement of abdominal wall compliance, fluid management, and improved organ perfusion [14, 20, 22]. As treatment, nonsurgical management is an important treatment option in critically ill patients with raised IAP [20]. In addition, many treatment options are available, and are often part of routine daily management in the intensive care unit (fluid resuscitation, the use of inotropes, positive pressure ventilation, nasogastric or enteral decompression tubes, enema, neuromuscular blocking, prokinetic agents, sedation/analgesia, body position, interventions that decrease fluid balance, and percutaneous catheter drainage) [10, 15, 20]. On the other hand, improved resuscitation and sepsis control decrease, but do not obviate, the need for open abdomen and progression for ACS [13, 17].

If conservative therapy fails, emergency laparotomy or colonoscopy is the most effective therapeutic approach to achieve abdominal decompression [1, 3, 4, 21–23]. In particular, surgical decompression with midline laparotomy is the standard ultimate treatment once ACS with organ dysfunction is established [13]. Thereafter, patients with an open abdomen require intensive care, and are permanently threatened by the quadrangle of fluid loss, muscle proteolysis, heat loss, and impaired immune function [22]. As a consequence, the complication rate increases dramatically after 8 days of open abdomen therapy [22].

An early sign of ACS may be a decrease in urinary output, and ACS patients often complain of abdominal pain and abdominal fullness [3, 12]. In addition, elevated IAP may induce adverse effects on pulmonary, cardiovascular, renal, splanchnic, musculoskeletal, and central nervous system physiology [4]. On the other hand, elderly patients sometimes develop dementia and experience reduced everyday activities that are common with aging, and they frequently complain of severe abdominal distension with hypotension, tachycardia, and tachypnea [3]. ACS progression can be life-threatening, with multiple-organ dysfunction in the respiratory, cardiac, renal, and gastrointestinal systems; therefore, the correct diagnosis of ACS onset and timely appropriate intervention are required for an optimal outcome [3, 24]. In this case, because of diabetic neuropathy, the patient did not complain of abdominal symptoms, therefore making it difficult to diagnose as ACS.

IAH/ACS may be prevented in patients undergoing laparotomy by leaving the abdomen open where appropriate [10]. If untreated, ACS can lead to multisystem organ failure and death, with nearly 100% mortality [1]. Additionally, ACS associated with new organ dysfunction or failure has a mortality rate of up to 60%, and despite many efforts, mortality in patients with ACS remains unacceptably high [1, 2, 8, 22]. The key to optimizing outcome is early abdominal closure within 7 days, because failure to do so increases morbidity, mortality, and fistula formation [8, 17].

In conclusion, we encountered a case of ACS due to extremely elongated sigmoid colon and rectum plus fecal impaction caused by disuse syndrome and diabetic neuropathy. The key message here is that clinicians need to keep ACS in mind as a differential diagnosis, and perform careful and detailed examination when encountering patients presenting with symptoms or risk factors of ACS. In addition, they need to precisely diagnose ACS and perform optimal treatment without delay.

## Abbreviations

IAH: Intra-abdominal hypertension
ACS: Abdominal compartment syndrome
IAP: Intra-abdominal pressure
